# Understanding factors that influence unintentional insider threat: a framework to counteract unintentional risks

**DOI:** 10.1007/s10111-021-00690-z

**Published:** 2021-10-28

**Authors:** Neeshe Khan, Robert J. Houghton, Sarah Sharples

**Affiliations:** University of Nottingham, Nottingham, UK

**Keywords:** Critical Decision Method, Cybersecurity, Human factors, Insider threat, Sociotechnical framework, Sociotechnical systems

## Abstract

The exploitation of so-called insiders is increasingly recognised as a common vector for cyberattacks. Emerging work in this area has considered the phenomenon from various perspectives including the technological, the psychological and the sociotechnical. We extend this work by specifically examining unintentional forms of insider threat and report the outcomes of a series of detailed Critical Decision Method (CDM) led interviews with those who have experienced various forms of unwitting cybersecurity breaches. We also articulate factors likely to contribute firmly in the context of everyday work-as-done. CDM’s probing questions were used to elicit expert knowledge around how decision making occurred prior, during and post an unintentional cyber breach whilst participants were engaged in the delivery of cognitive tasks. Through the application of grounded theory to data, emerging results included themes of decision making, task factors, accidents and organisational factors. These results are utilised to inform an Epidemiological Triangle to represent the dynamic relationship between three vectors of exploit, user and the work environment that can in turn affect the resilience of cyber defences. We conclude by presenting a simple framework, which for the purposes of this work is a set of recommendations applicable in specific scenarios to reduce negative impact for understanding unintentional insider threats. We also suggest practical means to counteract such threats rooted in the lived experience of those who have fallen prey to them.

## Introduction

The spread of internet-enabled services and devices into the workplace has led to significant gains in productivity and efficiency (Schuh et al. 2014). However, this technology also offers potential vulnerabilities for criminals, industrial saboteurs and extortionists to exploit, a matter of increasingly widespread concern and media interest (e.g., Dice 2020). Aside from what might be considered traditional hacking of digital systems at a technical level (e.g., Goethals and Hunt 2019), there is increasing prevalence of cyberattacks that require the unwitting participation of innocent individuals in terms of opening an attachment, clicking on a rogue link or otherwise inadvertently completing the last action that compromises a system (Verizon 2020). This innocent facilitation of cybercrime by insiders is considered a subset of “Insider Threat (IsT)” known as unintentional/accidental insider threat with the remainder of the category known as intentional/malicious insider threat comprising of deliberate and malicious actions carried out by disaffected or mercenary employees within an organisation (Mundie et al. 2013). Previous work in this area has tended to merge these two forms of insider threat and falls broadly into three areas of investigation: the largely technical including the potential for AI or machine learning to actively block threats (e.g., Morel 2011); the psychological which has considered whether personality variables can be associated with increased risk of an individual committing a cyber-breach either deliberately or accidentally (e.g., Hunker and Probst 2011; Hadlington 2018) and a more organisational approach that has tended to emphasise governance of IT systems in organisations and management practices intended to increase security (e.g., Cappelli et al. 2008, CERT 2013). Often technical, psychological and organisational approaches are combined to inform sociotechnical defences. Some contact has also been made between the nascent cybercrime literature and the larger and more established literature on safety and accident prevention (e.g., the application of Reason’s Generic Error Management System to the case of cyber-breaches, Liginlal et al. 2009) and attempts made to position insider threat within a sociotechnical framework (e.g., Nurse et al. 2014).

In the work presented in this paper we extend this sociotechnical perspective to examine unintentional insider threat specifically to investigate the individual and contextual factors that exist in the moment of an inadvertent cybersecurity breach: either sending email in a way that breaches data protection regulations or clicking on a malicious link in a received email. The approach taken was to conduct an in-depth exploration of decision factors at the time of the breach through an adaptation of the well-known Critical Decision Method (Klein et al. 1989). Whilst this method is normally associated with capturing expertise in decision making (e.g., with Fire Commanders, Pilots, Surgeons, Controllers, see Plant and Stanton 2013; Pauley et al. 2011; Bearman and Bremner 2013) our view of cybersecurity breaches here is that whilst viewed retrospectively as errors, they take place in the context of otherwise expert work that the method is useful in capturing the complexity of and indeed, in as far as cyberattacks rely on tricking people, may be considered the exploitation of expertise built around cyber breaches as experienced (rather than, perhaps, as imagined). Our aim in doing this is ultimately to offer a richer picture of why these events occur in order to design interventions to reduce their frequency or mitigate their impact. In order to position our approach, we begin by briefly reviewing extant literature on inadvertent insider threat in cybersecurity.

## Background

Generally, IsT can be understood as the vulnerability in systems and infrastructure pertaining to assets through the action or inaction of individuals or ‘insiders’. This vulnerability arises from individual’s access privileges and proximity to and knowledge of systems and a threat’s severity can be affected by insider’s skillsets and motivations. For the purposes of this work IsT and insiders are defined as follows:

Actions (encompassing skills, rules and knowledge-based behaviour) or inactions of individuals or groups wittingly or unwittingly that cause loss or harm to the security of an individual, organization or the larger society, without differentiating between cyber or physical  parameters. The individuals have authorized access (physical and/or cyber) to physical assets and to confidential information in order to perform a function for themselves or an organization, which results in compromised safety or a cybersecurity breach.

Based on insiders’ intentions two types of IsT exist: intentional (also known as malicious) and unintentional (also known as accidental) which can be posed by an individual or a group (Predd et al. 2008) and it is unintentional IsT (UIsT) that is of interest to this work. Unintentional insiders do not mean to harm the organisation, but their actions or inactions can put assets and operations of the organisation at risk, affecting systems’ confidentiality, integrity and availability (CIA security triad) and resulting in a cyber incident or breach. Examples of this are evidenced in real life through daily reports of successful ransomware attacks experienced by organisations where well-intentioned employees unwittingly interact with malicious content or accidentally exposed personal data.

### Technical defences

Technical defences involve software or algorithmic solutions that safeguard against cyber threats including IsT. Goethals and Hunt (2019) divide all cyberspace operations as offensive or defensive. Stemming from conventional security thought, Defensive Cyberspace Operations (DCOs) aim to defend against attacks. DCOs are further categorised as either passive or active cyber defences against threats. Goethals and Hunt (2019) list passive cyber defences as adopting best practice in setting up systems, system monitoring and exchanging information. Thus, passive defences would include measures such as configuration management, encryption (symmetric and asymmetric), configuration monitoring, data management (storage, access and architecture) and software updates. Passive defences can also include defences encompassed within Intrusion Prevention/Detection Systems such as anomaly based, signature based and stateful protocol detection (Magklaras and Furnell 2002). Active cyber defences involve actively defending against threats through intelligent interference but must be legally permissible in the country of operation. Active defensive techniques can include computer forensics (network and system based, mobile devices and email), Intrusion Prevention Systems (network based, wireless, network behaviour analysis and host based), cyber-physical systems, stateful protocol detection and anomaly-based identification (Zargar et al. 2016) and deception software (honeypots, decoys and address hopping). Both passive and active defences are used as solutions to guard against IsT through monitoring systems’ behaviour and containing the spread of malicious software in networks in the event of an attack. Popular research now includes developing machine learning algorithms and the use of AI for smart cybersecurity. Algorithm based solutions are popular and conventional in computer science and while these techniques can strengthen baseline defences such as those adopted in passive defences, active defences are not effective for guarding against UIsT alone due to its unintentional nature.

With concerns over technical defences being singularly focused on algorithmic elements numerous technical solutions have emerged that focus on human and process elements to identify insiders who might pose a threat (Ani et al. 2018). This is largely accomplished through considering individual psychological and behavioural characteristics. Such technical solutions can include detection of anomalies based on individual and group network behaviour (Legg et al. 2017; Agrafiotis et al. 2015; Chattopadhyay et al. 2018), background checks and rule breaking behaviour (Bishop et al. 2008; Greitzer and Hohimer 2011; Kammueller and Probst 2013; Ogiela and Ogiela 2012). While such solutions highlight the need for effective defences there are notable limitations that might arise from implementation. For example, the lack of professionally trained resources to assess psychological traits, targeting of individuals, creating a conflict environment at work and considering psychological aspects in isolation from other factors that exist in complex environments.

### Sociotechnical defences

Sociotechnical defences include technical, individual (including psychological traits) and organisational factors that influence IsT to design solutions for complex environments. Whilst there are numerous sociotechnical solutions being proposed we will discuss works that are the most relevant to safeguarding against unintentional IsT.

The National Cyber Security Centre (NCSC 2012) ‘10 Steps to Cybersecurity’ guide proposes a mixture of technical, individual and organisational defences that can in-turn guard against IsT. This guide includes PCDs such as network security, malware prevention software, secure configuration and managing user privileges. Individual factors include monitoring user activity and devices. Organisational factors include educational and awareness training for users based on security policies. It is recommended that trainings be regularly audited for effectiveness and staff should be encouraged to attain formal qualifications to build in-house security skills. Organisational factors also include communicating risks and acceptable use of company systems, regulating the use of removable media devices and an incident management plan that is strengthened through specialised training and rehearsed through drills. Organisations are also advised to promote an incident reporting culture that empowers staff to share poor practices without the fear of being blamed. While these suggestions are effective the guide also contradicts itself and states individuals should be held personally responsible for deviations in security policy as well as formal disciplinaries and actionable penalties that are enforced for IsT, which is effective for intentional IsT but gravely damaging for UIsT. Reprimands or penalties for UIsT can easily result in feelings of injustice, bitterness, embarrassment, blame, low morale and demotivation in an organisation.

One of the most popular and recognised frameworks is the MERIT insider threat model developed by CERT Program at Carnegie Mellon University’s Software Engineering Institute (Keeney et al. 2005; Cappelli et al. 2007, 2008). Their work involved evaluating case studies based on real world scenarios in interactive learning environments to mimic complex systems involving humans and technology. MERIT model is focused on evaluating insiders’ skills, motivations and opportunities to become an IsT. Determining the motivations of insiders includes individual psychological and behavioural profiling (including background checks). The framework also considers organisational characteristics and recommendations include awareness training, creation, implementation and maintenance of security policy, organisational interventions for insiders exhibiting concerning behaviour and managing employee expectations and various PCDs to secure systems and access points. CERT (2013) links UIsT to limitations in human performance and fallibility. Human errors would thus increase in likelihood with time pressures to deliver tasks, lack of knowledge, difficulty of the task and load on cognitive factors such as inattention. Limitation of this model are that individuals might not report concerning behaviour due to multiple factors that are at play in work contexts (Bell et al. 2019). It is also problematic to propose simplified solutions for a complex problem such as UIsT and viewing the system as decomposable to propose solutions in isolation from other parts (Hollnagel et al. 2015).

The combination of technical defences alongside psychological traits to identify UIsT has enjoyed growing popularity over the last decade with a range of psychological measures being adopted to detect UIsT (Hadlington 2018). A framework developed by Nurse et al. (2014) furthers CERT’s work discussed above. Similar to CERT, UIsT is associated to factors such as time pressures but these are positioned to stem from task objectives. Evaluating accidents in this format can provide insights into the root causes and allow corrective measures to be implemented. While this framework provides a better understanding of why errors occur, it has limited features to consider (not applicable categories) for UIsT and subsequently lacks concrete recommendations to tackle UIsT.

Liginlal et al. (2009) produced a framework for privacy breaches based on GEMs that includes an ‘error management program’ through examining errors. This program proposes looking at the root causes for errors, creating a defence-in-depth strategy that works to avoid, intercept and correct errors and periodically evaluating processes. It states that error avoidance emerges from poor feedback and lack of experience which can be overcome through training. Errors can be intercepted through better system design such as displays, monitoring and alarms. Errors can be corrected through conducting a timely investigation, an in-depth analysis of why the error occurred without blaming individuals and proposing solutions. Recommendations to strengthen organisational factors includes: careful consideration before implementing new systems as it can lead to new errors, developing explicit and effective processes to conduct tasks supported through training, individual’s resistance to change which can result in lower errors being reported which is strengthened through focusing on situations and systems rather than individuals and lastly monitoring work-related fatigue.

The last framework that is relevant to our work is developed by Greitzer et al. (2018) called ‘Sociotechnical and Organizational Factors for Insider Threat’ or SOFIT. SOFIT incorporates a combination of PCDs and ACDs, 271 individual behavioural and psychological factors (such as dark triad, dynamic states and personality dimensions) and 49 organisational factors to identify IsT (such as poor communication, inadequate training, ambiguous goals, stress, workload, blame culture, poor team management, poor system designs, environmental stressors, unrealistic deadlines, mismatch between expectations and abilities and morale). Limitations of this framework are the non-disclosure of the complete list of factors that are considered within each of the three categories, relying on individuals to report observed behaviours and non-disclosure of algorithm used as a black box to make assessments of potential risks.

The relevant technical and sociotechnical solutions discussed above propose defences from a standpoint of humans are the weakest link. When such a standpoint is adopted it is this human link that must be isolated, targeted and forced to conform should anomalies occur. Arguably technical defences promote solutions through a reductionist approach based on individual psychological or behavioural profiling. On the other hand, sociotechnical solutions consider a larger set of components in complex systems such as organisational culture and workload. However, they largely rely on reporting culture and an institutional entity that can surveillance procedural adherence and reprimand deviation instead of adopting a Joint Cognitive Systems approach (Woods and Hollnagel 2006). This policing on micro levels can be extremely challenging and simultaneously diminishes the importance of recognising factors that might come into play when complex tasks requiring cognitive resources are delivered in real world settings. In fact, research from a human factors’ perspective is only just starting to emerge that considers human errors in cognitive tasks (Canham et al. 2020) or organisational factors (Greitzer et al. 2018) to address UIsT or the loss of attention to some areas in dynamic events (Vanderhaegen et al. 2020).

Furthermore, the proposed solutions aim to address intentional and unintentional insider threats simultaneously with a singular focus that results in a ‘one size fits all’ solution. With the bundling of unintentional and malicious threats together the solutions result in being too severe for unintentional errors, self-contradicting or having limited applicability to UIsT, which is a limitation recognised by some of the authors themselves. In order to devise effective solutions that work UIsT must be examined separately to malicious/intentional IsT and afford individual ways of working within complex systems to enable human brilliance. We thus conducted a study to investigate how decision making occurs in complex cognitive tasks that resulted in UIsT being realised to identify factors that influence UIsT.

## Methods

To encourage a rich discussion around naturalistic decision making (NDM) when engaging with activities that resulted in cyber breaches, we used Critical Decision Method for Eliciting Knowledge (CDM) (Klein et al. 1989). CDM was particularly fitting for the design of our interview questions as it focuses on a major event retrospectively with probing follow-up questions to guide discussions. These probing questions assist in eliciting expert knowledge about how decision making occurs in cognitive tasks. While we recognise that individuals are not experts at falling for cyber attacks, the interest is that breaches happen in the context of expert behaviour at work or in our personal lives. CDM is also widely used across various domains to help analyse decisions (Hoffman et al. 1998) and inform system development and design. While CDM is normally used in homogenous samples (different individuals performing the same task in the same environment) we used this method differently as all our participants performed various jobs and worked on assorted levels in organisations for different periods of time with their employers. However, they all made critical decisions in complex work or personal contexts that led to all of them experiencing a major event of a cyber breach.

CDM begins with a general question about the incident and in this study it was the cyber breach to construct an initial picture of the incident from the participant. CDM then provides probing questions based on the information shared by the participant. The use of CDM to explore cyber breaches was valuable as it allowed participant and the researcher to journey into an introspective in-depth examination of the incident. It also allowed conversation to flow naturally and provide overall consistency across discussions. Inclusion criteria consisted of participants having experienced one of the three scenarios in their personal or professional lives: (1) They had accidentally sent sensitive information to the wrong recipient; (2) They had accidentally clicked a link that resulted in Phishing, Ransomware or gave someone access to their private information; (3) They had clicked a link by mistake that gave someone access to their email account, social media account, bank account or personal device such as a laptop or mobile phone.

Structured approach and emergent themes approach are usually adopted for data analysis to compliment CDM (Wong 2004). Structured approach assumes a pre-existing framework within which data is then coded and the emergent themes approach focuses on the relationships between concepts. Presumptions about data was a particularly significant factor in our selection of methods as we did not want to make any assumptions prior to analysis. We consequently applied a grounded theory (GT) approach. Originating from sociology (Glaser and Strauss 1967) GT has since become widely adopted method by researchers (Muller and Kogan 2010). Key points in the data were identified and assigned codes, known as open coding. Codes where then compared against each other in the same interview and across interview transcripts, known as constant comparison method (Hoda et al. 2010). This was done until data reached saturation before commencing analysis.

### Participants

Following ethical approval ten participants were recruited, eight from East Midlands and Greater London areas and two based internationally. Participants had to be over 18 years of age, have access to the internet and experienced one of the three scenarios in their personal or professional lives: (1) They had accidentally sent sensitive information to the wrong recipient; (2) They had accidentally clicked a link that resulted in Phishing, Ransomware or gave someone access to their private information; (3) They had clicked a link by mistake that gave someone access to their email account, social media account, bank account or personal device such as a laptop or mobile phone. Participants’ scenario of breach, settings (personal or professional) and their occupation at the time the breach are shown in Table 1. Participants had a varying degree of experience ranging from mid-level to advanced, shown in Table 6.

**Table 1:** Participant scenarios, settings and associated professional fields at the time of cyber breach

Participant	Scenario	Personal/professional setting	Field
P1	Accidently engaged with content that resulted in being hacked	Professional	Higher education (researcher)
P2	Accidently sent sensitive information to the wrong recipient	Professional	Healthcare (grants manager)
P3	Accidently sent sensitive information to the wrong recipient	Professional	Charity (grants manager)
P4	Accidently engaged with content that resulted in being hacked	Professional	Higher education (researcher)
P5	Accidently sent sensitive information to the wrong recipient	Professional	Charity (grants manager)
P6	Accidently sent sensitive information to the wrong recipient	Professional	Charity (international partnerships)
P7	Accidently engaged with content that resulted in being hacked	Personal	NA (student)
P8	Accidently sent sensitive information to the wrong recipient	Professional	Think Tank (internee)
P9	Accidently sent sensitive information to the wrong recipient	Professional	Food Retail (lawyer)
P10	Accidently engaged with content that resulted in being hacked	Professional	Charity (accountant)

Participants were not offered any compensation for sharing their experiences as part of this study and were provided associated materials describing the motives of the study prior to recruitment. As a result of snowball sampling some of the participants were known to the researcher (NK) in a professional context. Consent forms were completed and participants were given the opportunity to ask any questions prior to commencing any discussions.

### Data collection

Data was collected between March to April 2020. Discussions were held individually with participants and audio and video recorded, generating approximately 9.5 hrs of dialogue. Due to the Covid-19 outbreak in the UK, discussions were rescheduled to be held online on a platform most familiar to participants (Skype or Microsoft Teams) at a convenient date and time for them.

The full set of critical decision method based questions used in interviews are listed in Table 2. The researcher (NK) carried out discussions with participants and transcribed them verbatim from the digital recordings. NK also analysed and interpreted the data.

**Table 2:** Critical decision method based interview questions

Decision (initial question)	(a) Can you describe how you know if something is genuinely from the sender or not?
	(b) How do you think this differs from someone with less experience with technologies?
Knowledge (probe)	Where do you think you acquired this knowledge to differentiate between content that is genuinely from the sender and malicious content?
Experience (probe)	Thinking back to a specific time when you were cyberattacked, could you describe the incident from the time right before you received the malicious content/virus to the time after you had/were about to click the link?
Experience (probe)	Could you explain the sequence of events as they happened including how long each stage was?
Cues (probe)	What were you seeing, reading or hearing that suggested that this content was genuine?
Analogues (probe)	Were you reminded of any previous experience?
Goals (probe)	If things went according to plan, what were you trying to achieve during the time the incident happened?
Options and Basis (probe)	(a) Did you consider any other actions to take prior to clicking the malicious content?
	(b) [if applicable] How was this option selected and others rejected?
Aiding (probe)	What training, knowledge or experience could have helped to avoid clicking this malicious content?
Time pressure (probe)	On a scale of 1–5 (1 = no pressure; 5 = max pressure), how much time pressure was involved in making this decision?
Externals (probe)	Do you think other/personal goals impacted how you made decisions when interacting with what might seem to be malicious content?

### Analysis

**Fig. 1:**
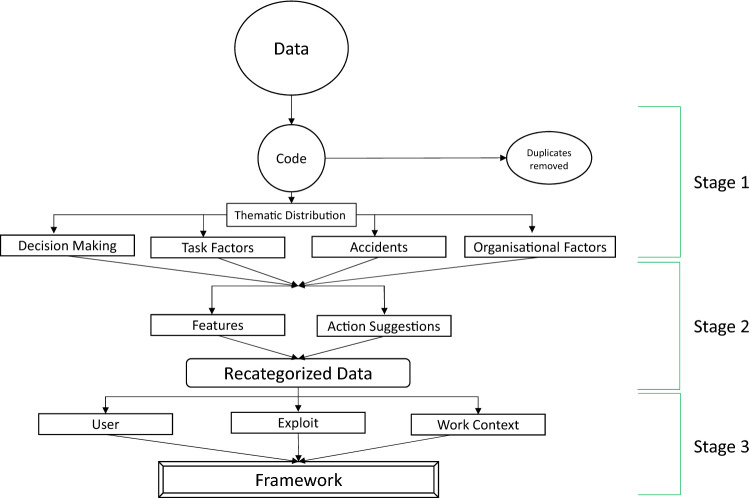
The three stages of data analysis

Transcripts of the digital recordings were produced, anonymised and uploaded to QSR-NVivo software for coding. Transcripts were highlighted with colour ink and descriptively labelled based on open coding and constant comparison method. Once labelling was exhausted to the point no new labels could be generated, a three-stage analysis was conducted shown in Fig. 1. In stage 1, labels were organised thematically as ‘Decision Making’, ‘Task Factors’, ‘Accidents’ or ‘Organisational Factors’. This thematic categorisation produced results that offered a better understanding of factors influencing UIsT. Stage 2 involved reorganizing codes as either features or actions. The results from this were used in stage 3 to further categorized according to the Epidemiological Triangle fields of ‘user’, ‘exploit’ or ‘work context’. From this recategorized data a framework was developed to list ‘inputs’ and ‘outputs’ that can be used by organisations to identify, intervene and mitigate against UIsT. For the purposes of this work a framework is a set of recommendations applicable in specific scenarios to reduce negative impact.

Data was analysed with a critical realist view (Bhaskar 1989) where the researcher believes that participants can offer insights to cyber breaches through their experiences and that researchers also play a role in constructing that knowledge. An overarching epistemological position of relativism (Siegel 2004) was adopted which believes that all knowledge generated is context specific to individuals, society, culture and time.

## Results

**Fig. 2:**
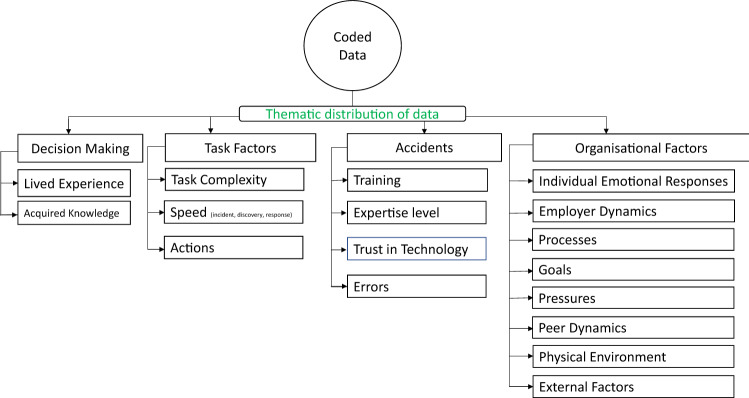
Thematic distribution of data

Four themes were identified in the analysis as factors that influence UIsT, presented in the following sections as: ‘Decision Making’, ‘Task Factors’, ‘Accidents’ and ‘Organisational Factors’, shown in Fig. 2. Numerous codes emerged from the data and code frequencies listed in Tables 3 and 4 allowed us to cross check the weightings of our findings.

**Table 3:** Total code frequency for each theme

Theme name	Code frequency	%
Decision making	340	25
Task factors	238	17.5
Accidents	238	17.5
Organisational factors	530	40
Total theme frequencies	1346	100

**Table 4:** Code frequency for subcategories within each theme

Parent category	Subcategories	Frequency
Decision making	Lived experience	188
Decision making	Acquired knowledge	152
Organisational factors	Individual	111
Organisational factors	External Factors	96
Organisational factors	Pressures	91
Organisational factors	Employer	88
Task factors	Speed (incident, discovery, response)	83
Task factors	Actions	80
Organisational factors	Processes	79
Task factors	Complexity of task	75
Accidents	Training	73
Accidents	Expertise level	61
Accidents	Trust in Tech	56
Accidents	Errors	48
Organisational factors	Peer Dynamics	31
Organisational factors	Physical environment	20
Organisational factors	Goals	14

Quotes from participants are identified as; participant 1 (P1), participant 2 (P2) and so on. All participants are referred to as ‘she’ and ‘her’ regardless of their gender identification to maintain anonymity. Various quotes from participants and their respective context which support the results are evidenced in Table 5.

**Table 5:** Quotes from participants in their respective context distributed thematically to support results

Theme	Sub-theme	Participant	Context	Supporting quote
Decision making	Lived experience	P8	P8, who experienced a cyber breach early in her career while working at a think tank in a metropolitan city, got visibly excited when sharing personal techniques for identification of malicious content. P8 saw this as a cat-and-mouse game where the hackers were always trying to outsmart unsuspecting recipients by impersonating real life entities and using misdirection to lure targets in. To stay a step ahead of the hackers P8 expressed the importance of paying attention and reading in between the lines	‘But if you look at the email [ID/Domain], it's usually some crazy weird email that they've just created. And they're trying to hide behind all of the email itself. Going on, you can also kind of tell that there'll be certain slight differences between the logos and stuff and the branding that they use. They might use an old, outdated one’
Decision making	Lived experience	P6	P6, who works for a charity organisation conducting extensive work overseas with a range of different partners, shared how she applies lived experiences to quickly validate the legitimacy of online interactions to support snap judgements	‘When it's from an address I do know, you know, that I recognise, if it's in a way that the person wouldn't normally sort of writes or interact with me, then that's big red flags there …. If it's like something that's general but specific, like, “Oh, that thing we did last?” but it doesn't give you the information of what exactly it is, it just points to something really quite general. Firstly, if that doesn't match up with anything in my experience with that person, then that's a no–no. But then even if it does, then you put on that, sort of, other lens of 'Okay, does this look credible?'’
Decision making	Lived experience	P4	Example with P4, who is a researcher in the higher education sector and experienced a cyber breach by interacting with malicious content that appeared to be from her supervisor. In this part of the discussion P4 shared how the lack of multiple channels to communicate a threat (similar to 2FA) assisted her in making a snap judgement	But if they were saying to me, "Oh, there's something wrong with your bank account, please log in here", I would more likely be like, 'Oh, surely I would have received a message' or, 'Surely I would have received a phone call'. So I'm more likely to contact the bank before I open stuff like that'
Decision making	Acquired knowledge	P4	When speaking to P4 about how she felt her knowledge was different to other generations P4 discussed the phenomena of fake news and how her position as a researcher allowed her to be untrusting of online interactions, which she did not feel was largely afforded to previous generations	‘Whereas I feel… they're[older generation] very much more different because I feel like this whole process of the internet was thrown at them without them getting taught, like, how to distinguish the truth from lies’
Decision making	Acquired knowledge	P2	P2, who experienced a cyber breach during an intense process of peer-reviewing grant applications in the healthcare sector, was pensive about the differences in knowledge between herself, her peers and other generations	‘I think I feel a bit more confident about making these decisions, but that's possibly misplaced. Because my parents, being less skilled are also less likely to click anything because they're terrified of making a mistake’
Task factors	Complexity of task	P2	P2 described peer-review stage as complex using various technologies to aid the task such as online searches, application portal, emails, excel sheets and PDFs. This entire process meant that while all application reviews started at the same time, the task was not synchronised and increased disproportionately in complexity as it progressed	‘So you're not paying attention to drafting just one email, you're doing a lot of things at once. And it's that kind of almost like sweet spot for disaster of being repetitive and dull, but still complicated. To make sure that the right thing is going to the right person’
Task factors	Complexity of task	P6	P6 discussed how adopting a systematic approach allowed her some control over a complex task that is not synchronized as it progresses	‘You've got a tonne of contracts to do. And you're scanning them and they're piling up on your desk, literally on the right-hand side and then you're trying to be systematic about the way how you're saving them. Putting the numbers there, the right codes there for the project reference numbers. Filing them in the right place and sending them on to the right people. But you're doing that at pace, probably…. You'd then go out to those people individually to ask them, you know how they wanted the contract to be set up… And so you're getting back those 45 different bits of information. And then I'd say that it's an iterative thing… And then you're trying to, one you're getting the replies in, your you're making the contracts, making them out to the right people, the right time, the right amount… But again, because there's 45 of them, they're all coming in and dribs and drabs and you're doing this process for each one’
Task factors	Complexity of task	P3	When talking about the complexity of the task P3 discussed how the human element was significant enough for her to mould the process in a way that made the human related aspects in the task run smoothly	‘So I think for everyone who agreed to review an application, we just sent all of them the PDF, as well as linking them on the system. Because it was common enough that people would prefer the PDF’
Task factors	Complexity of task	P9	In order to contest the fines P9 is responsible for filing a case in the local courts if her manager and the wider team approves. In the quote she describes the task that led to the cyber breach. Continuously interacting with new information was another attribute of the level of complexity for the task being delivered	‘Then we had the calls from both of the store managers giving a bit of explanations as to how much we were fined and exactly why we were fined. Immediately after that we had a Management Team meeting… and we're all discussing what happened. And then I'm told "Okay, file the case", okay, I'll file the case. And then I had a couple of immediate phone calls, right after each other, asking everyone for evidence. I got the evidence. And then I had an online team meeting with my internal audit team. And we talked about it[raid], we discussed everything that happened and I said, "Okay, my email", and I immediately go through all of my emails, I save all of the documents, all of the evidence, and I stick all of it in one email…Pictures, screenshots, emails (IV:Okay) Everything relating to the incidents and I emailed it to my internal auditor [[John1]] and copied it to my assistant [[Janette]] sent it off.’
Task factors	Speed of the incident, discovery and response	P3	P3 who was peer reviewing applications described the speed of the incident occurring over a few seconds	‘I think it was like, I think it auto-filled after I put in the, I started typing the first name. And it auto-filled the email address. And I just sent it off’
Task factors	Speed of the incident, discovery and response	P10	P10 is an accountant who accidentally engaged with malicious content that resulted in being hacked. Typical of our sample, P10 related the speed of the incident in the corresponding quote	‘I didn't realise at that time that I actually clicked the link. I realised that a week after. I probably clicked on it and then closed it straight away (IV:Yeah)’
Task factors	Speed of the incident, discovery and response	P5	P5 spoke of the speed of the incident and went on to say how she did not realise that a breach had occurred in the first place	‘And then I got an email back from somebody to say, "Hi, I don't think this was meant for me. What would you like me to do with it?". And it turns out that I'd actually sent it to another [[Joe2]] that I've previously emailed, that had come up automatically on my email list (IV: Okay)’
Task factors	Speed of the incident, discovery and response	P6	Speaking to P6, who manages overseas partnerships, described how it was her systematic approach to the task that allowed her to discover a discrepancy that identified a cyber breach which otherwise would have gone unnoticed	‘I had a separate spreadsheet where I was documenting which stage I was at with the [[funded]] projects. Mhm, and then I would've noticed the discrepancy between what I hoped to do [laughter] versus what I actually did. And so that recording[stage] would've been an immediate check’
Task factors	Actions	P10	P10 was doing a routine action when the breach occurred. Her comments highlight cues with contextual features that assist her in identifying if content is safe (such as an email from a known safe sender). However, in this instance, applying the same principles or the misapplication of the same contextual cues made her vulnerable to UIsT	‘It was an email from a company who we work with on a daily basis (IV:Okay) So I'm receiving emails from them quite regularly but at that time, I didn't realise that this email is actually a phishing email because it was sent from the person who I'm dealing quite often, quite frequently (IV:Yeah) I didn't think that email could be spam’
Task factors	Actions	P4	P4 shares her reasoning for progressing through what seemed like a usual request i.e., interaction with her supervisor. Making an assumption allowed her to validate an anomaly as she progressed through the decision making stage	‘And what it was, it was an email with, like, a picture of like a voice memo (IV: Okay) And I genuinely assume that my supervisor had just sent me a voice memo because I don't know, maybe that's his new way of communicating with me’
Task factors	Actions	P1	When speaking to P1, who is also a researcher in higher education and was hacked as a result of interacting with malicious content, she described her actions to contain the impact of the breach after discovering that she had been hacked	‘And yeah so, when the bank called me and I obviously killed the card and so on, and they blocked the transactions, so there's no harm done. But then I went to the [[Employer]] IT and they took it very seriously and… so everything was wiped out and reformatted’
Task factors	Actions	P10	Participants also undertook various actions to reduce the impact of the cyber breach which is highlighted in P10’s quote	‘And I remember he mentioned that he had to return emails too, but it was quite a large number of emails that had been sent from my email. So he[Head of IT] had sent to the people just to mention that my email has been hacked, so like an apologetic email just to reassure them that they should ignore this email and if they clicked on the links or other things, then it's better to change their password (IV:Yeah)’
Accidents	Training		P4, the researcher in higher education, shared her experience of training which was not seen to have much influence in deterring UIsT	‘So you know, obviously we receive these types of trainings (IV: Yeah) But it's hard to integrate them into real life’
Accidents	Expertise level, trust in technology and errors	P7	P7 spoke about the panic at seeing the ransomware notification (which appeared to be from the FBI demanding Bitcoin crypto currency) and thinking about the potential loss of emotionally valuable data such as digital family pictures. However, she was less phased by the malware itself. Analogue was absent (i.e., symbolic ideas of future steps to aid decision making that are drawn from lived experiences) but context present	‘I didn't really think it was a genuine notification because I read about it before that this is a malware’
Accidents	Expertise level, trust in technology and errors	P6	The experience of this incident was novel to a majority of the participants which is encapsulated in P6’s quote	‘…So yeah, I mean there would've been plenty of things that would've reminded me [laughter] but they wouldn't have been incidents. They would've been times when I caught myself before sending the thing (IV:Yeah) rather than breach’
Accidents	Expertise level, trust in technology and errors	P9	An example of analogue being present in P9's lived experience	‘Well I have done this a lot [(IV:Okay) laughter]. And the thing is, by that I mean, most of it might not be important… And then the irritation of having to fix everything. It's like I do a thousand emails a day, now I have to do five more just to fix this’
Accidents	Expertise level, trust in technology and errors	P7	P7's thoughts when exploring trust in technologies	‘But my installed antivirus software was a premium plan back then, so I was a little bit sad that it didn't work’
Accidents	Expertise level, trust in technology and errors	P1	P1's thoughts when exploring trust in technologies	‘There was nothing else. And those two things I think were definitely [credible]. Especially the fact that it came from someone from the [[Employer Name]] address. I mean, had it not been someone with a [[Employer Name]] address, I probably wouldn't have clicked on it. I think that was the main thing’
Accidents	Expertise level, trust in technology and errors	P5	Participants reported a distrust in technologies when collaborating on tasks shown in P5’s comments (who worked in a fast-paced environment funding grant applications)	‘So pulling up the data was, I think just pulling up the data in general… was [Online platform 2] at the time, was always a nightmare… Because when you pull up data, it just didn't. All of the comments weren't[ formatted], you couldn't read it[data] in a way that was easy to read. So it's up to me to format it and manipulate the spreadsheet to make it really easy to read for them[panel members]’
Accidents	Expertise level, trust in technology and errors	P1	Actions that triggered the cyber breach were seen as insignificant even as the incident unfolded and its overall significance underestimated as seen in P1's comments	‘So that wasn't very good judgement from me at all [laughter] I was like, “Oh, whatever”. And I carry on as per usual, you know… Then then after that, because in my mind after that, once I discovered the document, and I was like, “Okay, this must have been an error”. And then I didn't think back’
Accidents	Expertise level, trust in technology and errors	P4	When speaking to P4, a researcher in higher education, she discusses how after clicking the malicious link nothing happened (lack of feedback loop). She made an assumption about an error being made	‘I remember emailing him to say, “Oh, like I'm not able to download what you've sent me. Can you send it to me again?” (IV:Yup). So I must have genuinely have been believing this through the whole timeline of it’
Organisational factors	Individual emotional responses	P10	P10, the accountant, described how the malicious virus was activated a week after clicking a link from a known client she had frequent contact with. She described receiving a phone call initially from another client to alert her about a suspicious email they had received from her account. Whilst she’s on the call with them her emails and phone lines get inundated with other clients calling her to alert her or to confirm if the email is genuine. P10 goes on to share her emotional response which is exhibited in the corresponding quote	‘And at the same time I was feeling really embarrassed because of the way I was acting in clicking on this email (IV:Yeah) I should be more considerate and [laughter] more observant in a situation like this’
Organisational factors	Individual emotional responses	P2	P2 sharing her emotional response to discovering a cyber breach	‘So yeah, there was obviously that like cold dread realising like, 'Oh, that really shouldn't have happened'’
Organisational factors	Individual emotional responses	P6	P6 sharing her feelings of disbelief of being a victim to cyber attack	‘What happened when I did realise was a big "Arghhhh!" moment. Just like quite a quick, mhm, I mean I got on the email very quickly to ask them to discard it… Yeah so I mean I was aware of the pitfalls and I thought I had robust enough personal systems to deal with it. But of course not in this instance’
Organisational factors	Individual emotional responses	P8	P8, who worked at a think tank in a metropolitan city when she experienced a cyber breach by sharing sensitive information that also resulted in her account being hacked, shared her feelings at the time of the incident	‘A lot of panic, anxiety (IV:Okay) I wasn't so much surprised. But you know when you've done something wrong, and you have this immense feeling of guilt, and you're like, "Ah, no!", and you just want it to go away. And there's nothing that you can do, you're powerless to do it, because you've already done it and there's nothing you do now, but own up to it’
Organisational factors	Employer dynamics	P10	P10, the accountant, discusses the long-term response from her employer as a result of cyber breaches that were occurring at the company around the same time she was compromised. P10 states how IT have instated an automated notice to emails that are received	‘But recently IT what they [do], any external emails which we receive there was a warning… "Do not open links or attachments unless you trust the sender and know the content is safe". We sort of, they, after all the incidents at the company they decided to do it the other way. I mean to protect the staff (IV:Yeah)’
Organisational factors	Employer dynamics	P2	P2 who worked in healthcare funding discussed a covert penetration testing conducted by an external consultant firm. P2 mentions the impact of the message which appears to elicit desirable behaviour through punitive measures	‘So this happened over the course of several months, and then they did a big presentation basically about how terrible we've all been and how we'd failed the company in many ways [laughter]… We couldn't really add in more time [to the deadlines], never can. Well, sometimes you probably can, but that never seemed to be the option that came up… So, yeah, there were changes that that came about anyway. So you kind of get the more direct ones were like the checklists and things that we partly used as training for new people and just to have documentation. The company actually had like an ops team and policy documents that everyone had to sign, you had to read them and have like an Ops[Operations] induction and testing and data protection training and all that stuff’
Organisational factors	Employer dynamics	P6	P6 discussed the responsibility placed on individuals if things went wrong—a classic example of blame culture	‘But then what comes with that money is resource constraints. So you can't spend that much on staffing. And so that[incident] was definitely you know [that time], at particular crunch points like the end of year where you have quite a lot of activity happening. You spend the rest of the year planning for and then sort of implementing and then it comes down to quite a lot of administrative stuff whilst also delivering on top of your day job all like in this kind of period without really additional resource being there. So I guess that's where the funding side comes in. There's constraints that come with it, mhm, that force to you deliver sometimes and give you much more constrained time frame in which to do so… And without too much kind of, I don't know how to say it, at that point mm, it wasn't like a facilitative environment, it was more if something goes wrong you need to [laughter] have somebody to blame [laughter]’
Organisational factors	Employer dynamics	P5	P5 who worked in a fast-paced grant environment reportedly had an open and approachable relationship with her line-manager and promptly reported the cyber breach as soon as she discovered it	‘I went to [[Joe1]] my line manager and asked him what I should do. He[Joe1] went to [[Jane]] who was our [[Head's Designation]]. And [[Jane]] came up to me and firstly asked me to ask [[Joe2]] that I had sent it[email] to, to delete it. So I did that. And then once [[Joe2]] replied to me to say that he had deleted it, that was kind of just the gist of it really (IV: Okay) Which lead me to getting a stern, "So, please don't do that again" [laughter]… [[Head of Ops]] would be responsible for that. He would be responsible for processes and procedures (IV: Okay) so making sure that they[policies] were being kept I guess’
Organisational factors	Employer dynamics	P10	This comment showed that there were tasks that took P10 longer than it would others. For such instances she had to inform management when she was engaging with such tasks, reflecting how time might be a limited resource within their team and require advance planning	‘If you are asked my Director, it's gonna be very easy for him [laughter] Because he's got that extra knowledge in Excel [laughter] Sometimes I struggle so, for me I have to do for example, the day I have to do reconciliation for something and it took me a bit longer so I needed to get in contact with him’
Organisational factors	Employer dynamics	P8	P8, who worked at a think tank, reflected on the consequences of the cyber breach reflecting a lack of organisational and individual learning	‘IT [department] just shut down my account and then they made me a new account. And that was it and it was like "Carry on, start again". And I don't remember there ever being like, I was ever told off, I was never given a lesson on what I did wrong, except for the fact that BCC not CC’
Organisational factors	Employer dynamics	P2	P2, who worked in healthcare grants, also said that she did not encounter any real learnings from the experience	‘Obviously, I suppose they don't want to come down too heavily on people because they do still want you to say that this has happened. So I had to apologise to the person and ask them to permanently delete the email. And then obviously find new reviewers and make sure that I follow the process more closely in the future’
Organisational factors	Processes	P2	P2, who worked in healthcare grants, reflected on the prescribed processes, reflecting her experience level	‘So they[Previous Employers] were quite good in that sense that they had a lot of things written down already, but not often the nitty gritty of how you do things and why it was important to not skip the step or whatever’
Organisational factors	Processes	P6	P6, working with overseas partners, discussed how she enhanced processes in each cycle, reflecting her experience level	‘That was probably my second time through it[stage] and the first time, again, similar thing, everything rushed, lots of things going on. It would’ve been a lot of misses, right? So that’s why you sort of develop these systems which you try to become more robust with [laughter] Or you just remember where the near misses were. And then either develop systems or just be very, very weary and then give yourself proper time to be able to focus. I think that’s the main thing really whenever you’re engaging with something quite so process orientated’
Organisational factors	Processes	P3	P3, who was peer reviewing applications for scientific grants, discussing processes that reflect her experience level	‘I should say actually, in fairness, this process wasn’t really how things were supposed to work. Like, the applications were supposed to be sent securely through the grants [online] system… So, yeah, it wasn’t good practice I would say and even within the processes as they were set out… But also, yeah, then they have to do the reviews. And some of them would do it through the system and so they would come in automatically. And some of them will email it to you. If they were people who didn’t really like engaging with the system as much’
Organisational factors	Goals and pressures	P5	Speaking to P5 who worked in a fast-paced environment, she discussed her motivations, challenges and pressures at the time of breach	‘You have a deadline of when your award meeting is, but there’s so many kinds of things that come up along the way, that just delay you (IV: Yeah)’
Organisational factors	Goals and pressures	P6	P6 who worked with overseas partners discussed her motivations, challenges and pressures at the time of breach	‘So it was quite pressurised only because, mm [thinking pause], like there’s a lot, because you’re under pressure to hit the financial deadline but a lot of the process to getting there isn’t in your control necessarily (IV:Ok)’
Organisational factors	Goals and pressures	P1	P1, the higher education researcher, elaborates influencing factors beyond time pressures	‘But it’s just if you do these things in a rush, I think, and you don’t take your time to even read the URL, so, you might just do it kind of instantly… And I had a deadline coming up in a couple of weeks. So I was in, like, not stressed as such, but kind of in a rush’
Organisational factors	Goals and pressures	P10	P10, the accountant, also elaborated on the settings of her cyber breach that demonstrate influencing factors for UIsT	‘I remember that because Mondays and Tuesdays, they’re always quite busy for my workload, because I have to make sure that all the payments are prepared and everything has to be processed before the end of the week… You know, when you’re in a rush and doing things you could just accidentally, without really thinking, you’re just going through and because you’re in a rush to do other things you don’t really focus on what you’re clicking’
Organisational factors	Peer dynamics and physical environment	P9	Participants discussed relationships with their peers before and after the incidents as well as their physical environments. P9 reflected on the impact of the breach on her peer dynamics. Quote also exhibits how she had to justify assigning a different person to the task which was discovered by the unintended recipient as a consequence of the cyber breach. This quote also shows undercurrents of potential friction caused in the relationship	‘The negative repercussions of this are, is that (a) my lawyer [John2] knows that I didn’t give him two cases and someone else got them and he’s feeling a bit antsy. Because he called me again later and he was like, “You didn’t give me those cases”. And I was like, “Yeah, I didn’t want to. I gave them to someone else”’
Organisational factors	Peer dynamics and physical environment	P8	P8’s comments reflect a sense of alienation from her peers during the cyber breach which otherwise appear to be good inter-relations at a peer-to-peer level	‘But within my immediate group, everyone was quite quiet, because they were doing their own work and doing their own thing. And I was just slowly panicking [laughter] Being very quiet because I didn’t know what to do, like, I told my manager… As everyone else was just doing their normal thing, living their normal life, being their best them, like doing their job’
Organisational factors	Peer dynamics and physical environment	P6	P6, who worked with overseas partners, as described her relationships with peers in the context of the cyber breach	‘I have a colleague sitting right by [me] and I would’ve explained something [laughter] “I just sent the wrong contract to the wrong people”, maybe had gotten a little bit of advice of how to go about it or something like “Don’t worry, happens all the time, it’s okay” that kind of thing. And then gotten on to try and correct the error pretty promptly (IV:Yeah) after that. But yeah’
Organisational factors	External factors	P6	Participants shared certain traits that might have contributed to UIsT, such as those exhibited in P6's statement who managed overseas partnerships	‘Ideally what I like to do is as soon as I'm getting something back, sort of send it out as quick as possible. So once I know, then I'd quite like to have things go and be in process (IV:Yeah) as early as possible (IV:Yeah)’
Organisational factors	External factors	P8	Participants shared certain traits that might have contributed to UIsT, such as those exhibited in P8's statement who worked at the think tank	‘So like, there wasn't any pressure on me to get this email out. But an eagerness for myself to do a good job, which is the irony of it all’
Organisational factors	External factors	P9	Participants shared certain traits that might have contributed to UIsT, such as those exhibited in P9's statement who worked at a Food Retailer as a lawyer	‘And this is not just a job, it's a really good job… That is the number one pressure that's been in my head for the past four months. Not to slip up, not to do anything silly, to make sure that I'm doing the right thing, that I'm appearing to be as efficient and competent as I hope I am’
Organisational factors	External factors	P3	P3, who also funded scientific grant applications, listed another factor that might have influenced UIsT. This snippet from P3 shows a tension between prescribed processes and factors that might influence deviation	‘While you do connect them through the system, you also send them a PDF, just so they can have something that they can read on the plane or something. Without faffing around with logging into the system, etc. So, yeah, it wasn't good practice I would say and even within the processes as they were set out’
Organisational factors	External factors	P6	Self-esteem as another factor that participants were actively considerate of. This included how they appeared to themselves and others around them as shown in P6's comment	‘And this thing[program] it was one that I had personally developed too and it didn't, so you just want to not be the [let down]’
Organisational factors	External factors	P2	Self-esteem as another factor that participants were actively considerate of. This included how they appeared to themselves and others around them as shown in P2's comments	‘But it wasn't kind of direct competitive at the time, but it's like you're aware of what you're [doing] and what everyone else is doing because you're all doing the same task. So you don't want to be the one that's falling behind. Particularly if the team is understaffed, you're having to pick up slack. So there was probably some aspect of not wanting to look bad. Not only because I was new, but because I was as new as other people, we were doing the same stuff. Like not wanting to look bad within that group setting’

### Decision making (DM)

All participants were asked to reflect on how their lived experience and acquired knowledge affected their DM when interacting with technologies. This was interesting as it gave an insight on how individuals might make informed decisions when identifying between malicious and non-malicious content that included utilising cues and applying knowledge-based behaviour. Cues incorporated surface features (logo disparities or brand colours) and contextual features (typically around pre-existing expectations around who respondents would expect to hear from and the nature of their likely requests) which we discuss below. This theme was divided into two sub-categories: lived experience and acquired knowledge. The ‘lived experience’ category comprises of direct personal experiences which might contribute to ‘acquired knowledge’. Acquired knowledge encompasses all channels used to build knowledge which can include lived experiences as well as other channels such formal classroom teaching, word-of-mouth or awareness campaigns.

#### Lived experience

All our participants shared a sense of reliance on their lived experiences which formed tacit knowledge to help them differentiate between genuine and malicious content, each with their own set of techniques and strategies to serve as defences. Techniques included clues from technical elements of the interaction such as the legitimacy of embedded web links, sender domain, font and logos. Participants also mentioned deviation in language and errors in the main body text to identify malicious content. Apart from the techniques used to evaluate technical elements of emails, participants also shared techniques that assisted them in making snap judgements about whether something was malicious. Techniques included reference to an incident that didn’t occur or if something was too good to be true which reflected a mixture of Type 1 (intuitive thinking which is rapid and autonomous) and Type 2 (reflective thinking which requires working memory and other resources) thinking processes (Evans 2012) at different stages in their interactions. Having specific context to a conversation, the nature of the request from the sender and relying on a strategy similar to two-factor authentication (2FA) from establishments such as their banks also assisted them in their DM. While relying on 2FA approach to validate false alarms may appear to be a relaxed state, P4’s technique reflects that her lived experience where no action was taken has resulted in things continuing safely. Interestingly, this strategy might aid in counteracting malicious emails that use urgency or time pressure techniques to lure targets.

#### Acquired knowledge

Participants largely appeared to have more confidence in their abilities compared to older generations specifically based on how various platforms were utilised whilst acknowledging that this confidence could be misplaced. Participants also considered informally acquired knowledge as more advantageous when safeguarding oneself against malicious content. Based on their personal and professional experiences all participants believed that they were comfortable with using technologies due to their exposure of growing up with it. This familiarity with technologies also brought a heightened risk awareness amongst all our participants for susceptibility to being scammed themselves and difficulty in identifying sophisticated scams. However, this heightened risk awareness might have stemmed from all our participants experiencing a cyber breach and not necessarily an attribute of comfort with technologies in general. Knowledge of cybersecurity was largely acquired through personal experiences, proactive online researching and social networks (online and offline). Some participants also mentioned other avenues such as classroom/lab based teachings and/or posters, marketing leaflets and bank app notifications that contributed to their knowledge but noted that these were less effective.

While lived experience and acquired knowledge help guide DM in our daily lives, the techniques deployed by individuals can subtly contribute towards indicators for assessing UIsT risk levels. If individuals are aware of latest techniques used by attackers, are confident with the use of technologies deployed to perform daily tasks or have internalised techniques to help identify malicious attempts it can provide a strengthening of defences (which we will discuss later in the framework). Equally, if individuals exhibit over-confidence or low levels of malicious content identification techniques (such as those discussed above) it might indicate a weakness in defences as end users would not possess the skillsets needed to make critical decisions in daily tasks that can result in UIsT being realised.

### Task factors

Broad themes that directly linked to task factors in the context of the incident of a cyber breach included complexity of the task, speed (of the incident, discovery and response) and actions (to minimize impact, conclusion assumption and subsequent actions).

#### Complexity of task

Almost all participants reported tasks as being complex with many preceding or simultaneous actions that needed to be performed in order to successfully complete the task at hand. All our participants described using a mixture of techniques to deliver their respective complex tasks that included using templates, manually maintaining progress spreadsheets, pivot tables, e-mail merges, performing manual checks for accuracy, searching the internet, collating information, bespoke software platforms and various mass market software.

Beyond the use of technologies, participants frequently mentioned the human element that informed and influenced their task delivery. The human element was significant enough for participants to mould the process in a way that made the human related aspects in the task run smoothly. Since work is not conducted in a vacuum or in a linear way, all the tasks involved other people contributing in some way but almost all of the tasks involved other people actively feeding-in (internal and/or external) for the successful completion of the task. This collaboration with people appeared to influence participants’ actions for how the task was conducted.

The active feeding-in from other people for the delivery of the task also created new information that needed to be processed and managed by the operators before future steps were selected, adding another layer to the complexity of the task. Participants interacted with information in numerous ways such as knowledge building, dissipating information to other people/systems and/or collecting information to inform their next step in the process.

Interacting with elements discussed above such as technologies, people and information contributes to the classification of complex tasks. In our study the delivering of such complex tasks facilitated new ways of working or in cases where processes were prescribed they were deemed insufficient in light of new information. We thus concluded that the complexity of a task can contribute to UIsT and processes should routinely be evaluated across all designations as indications of potential UIsT which we discuss in our framework later on.

#### Speed of the incident, discovery and response

While participants were engaged in delivering various complex tasks, unsurprisingly they all reported a very small window of time (seconds, minutes) over which the cyber breach occurred. The speed of the incident itself was so fast that some participants did not initially recognise they had experienced a cyber breach at all. Participants’ experiences reflected how users at any given time are a-click-away from a cyber breach occurring which might be a feeling that was heightened in our participants post experiencing a cyber breach.

Not identifying if a breach had occurred straight away was common amongst our participants whilst the time taken to discover a breach varied (some cases it was within a minute, some took up to a week). The discovery of a breach was reliant on either on some form of checking process or delayed feedback. Examples included the use of checklists, other people identifying an anomaly with participant’s account or a pop-up from the malicious software itself. In many cases this feedback was delayed and serendipitous suggesting an absence of clearly identified feedback loops and the possibility that some breaches may remain undetected for an indeterminate period of time if they are detected at all.

Once the cyber breach was discovered participants described a quick response speed to protect their cyber defences. This time window varied between a few minutes to a couple of days but in all cases was longer than the time window for the incident to occur depicting a typical gulf of evaluation (Norman 1986) for unintentional cyber breaches.

As the incident was so rapid in its nature and participants were performing complex tasks, we discuss how this element contributes to its fluctuating nature of UIsT which must be evaluated. We also discuss how assessing tasks to evaluate adequate feedback loops is essential and processes should be in place where individuals are familiarised with protocols in the event of a cyber breach to respond to threats promptly and effectively. Later on, in the discussion we introduce stop, think, ask, action, consequence or STAAC as a heuristic means of introducing reflection on actions taken.

#### Actions

Participants shared the actions they took prior to and during the breach. Contextual features appeared to assist participants in identifying safe content (such as an email from a known safe sender). However, applying the same principles or the misapplication of the same contextual cues made participants vulnerable to UIsT (i.e. receiving a virus from a known safe sender as their account had been compromised). This misapplication of contextual cues was often supported by assumptions being made to validate anomalies in interactions with people and systems in turn increasing the susceptibility to UIsT as users progressed in their DM to engage with malicious content.

Our results showed that during or immediately prior to experiencing a cyber breach, participants formed assumptions or had contextual cues that encouraged them to continue progressing with the task or underestimate the impact of a potential cyber breach. In all cases participants carried on with their normal duties until a cyber breach had actually been discovered. In contrast to not knowing if a cyber breach had occurred, once participants discovered that a cyber breach had taken place, they all performed some form of action to reduce the impact of the cyber breach. These actions included turning off equipment, reporting the incident to managers, contacting IT specialists and recalling the message.

Thus, actions can become critical for how to avoid or tackle a UIsT should it be realised. Findings from this heading feed into our framework by assessing individuals’ understanding of outcomes from a cyber breach and ties in with a culture of empowerment which we discuss in Sect. 5.

### Accidents

The broad themes that directly linked to the incident of cyber breach itself included training, expertise level, participants’ trust in technology and errors (expecting errors from others, error in expectations and accepting errors in themselves).

#### Training

Participants were also asked if training could have helped them bypass the cyber breach. In our sample training was not seen to have much influence in deterring UIsT. While participants believed that training was slightly useful for general and theoretical awareness, they did not feel it could be good enough to bypass the cyber breach especially given the unintentional nature. Reinforcing earlier findings, participants identified informally acquired experience as more potent with all participants identifying sharing of knowledge with peers as valuable. Peer-to-peer sharing of knowledge and experiences through any medium (face-to-face, emails, forums, trainings) was believed to be a more effective form of learning and awareness than training for avoiding UIsT. The experience of a cyber breach appeared to have the most influence on avoiding future UIsT amongst participants. Our results indicate a direct correlation between UIsT and personalised experience, where first-hand experience can be the biggest deterrent, followed by experiences of people that are known to you in real life and training being deemed the least effective. We incorporate this finding in our framework and discuss this in greater detail in our discussion below to recommend personalised trainings that are audited and instilling a culture of empowerment to help mitigate UIsT.

#### Expertise level, trust in technology and errors

All participants identified themselves as having mid or advanced level of expertise at their jobs, listed in Table 6. Eight participants shared that at the time of experiencing the cyber incident they were not reminded of similar lived experiences in the past, which are known as analogues to help aid decision making. Analogues were absent in instances where context was present for the participants (for instance being able to identify a popular ransomware’s pop-up but not having similar lived experiences). Similarly, some participants shared that they had context as an analogue which meant that their lived experiences either encouraged them to proceed in the task despite reservations or identify the threat as it was unfolding (early detection). Analogues also included recalling previous cyber incidents, but none had escalated to a cyber breach. Overall, the experience of this incident was novel to a majority of the participants. In fact, for two of our participants analogues were present and it aided them in identifying subsequent steps to take moving forward and/or anticipate consequences.

**Table 6:** Participant settings, levels of expertise and presence of analogues

Participant	Cyber breach setting	Novice	Mid-level	Advanced	Analogue?
P1	Professional		x		Absent but context present
P2	Professional		x		Absent
P3	Professional			x	Present
P4	Professional		x		Absent
P5	Professional			x	Absent
P6	Professional			x	Absent
P7	Personal		x		Absent but context present
P8	Professional		x		Absent
P9	Professional			x	Present
P10	Professional		x		Absent

When discussing the cyber breach and exploring trust in technologies, participants had trust in technologies to protect users from harm such as malicious content blocked by firewalls. Participants also largely described a trusting relationship with technologies for automated elements within tasks. This included not cross-checking automated actions such as recipients that are auto populated for emails or not suspecting emails from within the organisation. In fact, this very trusting nature for systems to be secure resulted in a majority of the participants being victims to a cyber breach.

However, participants reported a distrust in technologies to perform a task correctly which included concerns such as manually cleaning data when exported to make it readable, reliability issues with exports, user problems such as early time-outs and forgetting passwords, all of which led to human input to overcome software limitations. Human input also led to participants making assumptions during various points of the cyber breach. For instance, actions that triggered the cyber breach were seen as insignificant even as the incident unfolded and its overall significance underestimated.

Assumptions were also made about various elements of the cyberthreat by some participants. This meant that interactions that seemed out of the ordinary were normalised by the participants as they could associate a reason for why the interaction was occurring. Lack of a feedback loop allowed participants to assume there was an error (for instance a broken link, wrongly attached file or an error in the process or human error) but only after they had been compromised.

Expertise levels can result in greater levels of analogues being present when deciding how to react to potential or unfolding UIsT. Analogues can assist in effective steps taken to contain the threat and are incorporated in our framework under evaluating automated tasks, assessing technical skill levels and evaluating effectiveness of guidelines in the event of a cyber breach. Individuals’ trust in technologies is also a notable factor contributing to UIsT and this is addressed in our framework through evaluating software limitations and evaluating levels of trust in technologies amongst employees to strengthen defences. We incorporate errors in our framework by evaluating individuals’ ability to question, share and challenge abnormal interactions that would indicate a culture of knowledge sharing and empowerment. We discuss these elements in greater detail as part of our framework below.

### Organisational factors

Another major theme that emerged from the data involved factors that related to the wider context under which participants performed their tasks, relating to organisational factors. Sub-categories included individual emotional responses, employer dynamics, processes, goals, pressures, peer dynamics, physical environment and external factors all of which appeared to interplay with the conditions that facilitated an unintentional cyber breach.

#### Individual emotional responses

A range of emotional responses were shared by participants during the interview following the cyber breach. Overall, there was a feeling of disbelief that the incident happened to them which elicited feelings of embarrassment and gullibility making them more cautious going forward. Participants shared having felt guilt and a sense of personal responsibility for being compromised. Furthermore, participants also shared feelings of frustration at themselves, software and processes that facilitated the cyber breach. As participants developed a level of caution post the cyber breach, we incorporate measuring levels of caution amongst employees to assess user vulnerabilities for UIsT, which we discuss later on.

#### Employer dynamics

Discussions at the interview also included employer’s response to the cyber breach which was interesting as it shed light on some of the organisational factors that might have contributed to the UIsT incident that participants experienced.

Organisations’ actions following a cyber breach appeared to fall short of strengthening cyber defences against UIsT. For instances in our findings, one example is a disclaimer ribbon on emails by the employer for employees to only interact with content that they recognise as safe. While this prompt can be a useful reminder it would not have prevented the UIsT experienced by P10 who recognised and trusted the sender. Beyond this trust, it would be problematic to know if the content was safe without exploring it as the malicious link did not have any identifiable anomalies to P10 i.e. it did not appear malicious. These types of notices can create a safety climate (Neal and Griffin 2004) as opposed to a safety culture (Reason 1998) and be seen to place full responsibility on individuals for their actions in-turn propagating a blame culture. Overall for our participants, where applicable, IT department personnel helped to combat the threat without placing blame but there were undertones of how the incident created more work for them. IT’s countermeasures caused participants to experience downtime which disrupted their work with additional follow up tasks such as password resetting.

Participants also discussed their relationships with their line managers as well as any senior designations that were involved once a cyber breach had been identified. P2 mentions the impact of the employer’s message which appears to elicit desirable behaviour through punitive measures. While measures such as checklists and improved processes can be a step in the right direction, in P2’s case it is implemented in a way that placed responsibility on individuals if things went wrong—a classic example of blame culture where humans are seen as the weakest link in systems. This blame culture within organisations was also echoed in other participants’ accounts.

A majority of the participants shared how they were able to work autonomously with approachable managers. This open communication correlated with participants’ willingness to share the cyber breach with their managers early in the lifecycle of the threat. Participants also expressed having good immediate relationships with their peers and managers. However, participants’ experiences beyond these immediate relationships largely reflected a blame culture discussed above. Additional tasks that were introduced by employers as a result of the cyber breach to safeguard against UIsT would be fundamentally inadequate, such as signing additional contractual documents or being told not to do that again. All our participants shared the sentiment of limited resources to perform their tasks (such as time and people) in the organisation which was believed to be a contributing factor to the cyber breach.

Overall, in all our participants there appeared to be a lack of organisational and individual learning and accountability from the cyber breach. We incorporated this finding in the framework by evaluating the effectiveness of prescribed processes. Findings from Employer Dynamics also contribute towards evaluating the effectiveness of guidelines in the event of the cyber breach, assessing individual’s understandings of protocols in the event of a cyber breach, evaluating relationships between individuals and their managers, assessing stigma associated to incidents, levels of organisational communications about cyber incidents and assessing resources available to deliver tasks, all of which are discussed in greater detail in Sect. 5.

#### Processes

Participants described vague processes in place that generally guided them in how to perform various tasks. All our participants were relatively experienced in performing the tasks at hand and discussed how this familiarity allowed them to skip steps in the process that they did not deem important. Skipping steps or using unofficial channels was linked to saving time, efficiency or convenience, indicating established routine violations (Reason et al. 1990). Participants also discussed how processes had limitations, how their existing context facilitated their error but more importantly how they were aware of processes having limitations or potential for errors if followed as prescribed. These findings contributed towards the input of evaluating effectiveness of prescribed processes amongst skilled staff in our framework.

#### Goals and pressures

Discussing participants’ goals at the time was important as it reflected their motivations for the task and how they performed it which might have contributed to the UIsT they experienced. When asked to declare time pressures experienced on a scale of 1–5 participants reported feeling under time pressure to deliver the task with an average score of 2.8 points/participant. Pressures did not solely emerge from time constraints (time pressure listed as 2.8 on a five-point scale) but also from deadlines and feeling a lack of control. Furthermore, participants also added other goals that motivated them which included: wanting to move on to another task, following the prescribed process, desire for a lower workload after successfully completing the task and being able to achieve a larger more important goal through the completion of the task at hand.

Stemming from this discussion about pressures participants went on to elaborate factors that were at play at the time of the incident. Participants elaborated on other factors beyond time pressures and experiencing factors such as planned deadlines and anticipated workload led to participants wanting to move on and rushing which in some cases was supported through implementing automatic behaviour to progress through the task. Findings from this theme contributed to the input of assessing individuals’ motivations when delivering tasks to assist in identifying potential UIsT.

#### Peer dynamics and physical environment

When understanding the context in which cyber breaches occurred participants discussed relationships with their peers before and after the incidents as well as their physical environments. Overall, all our participants generally described having a friendly relationship with their peers which included being able to openly communicate with one another for advice and provide support through the cyber breach. They also described having a competitive relationship with their peers and everybody working autonomously to deliver their individual key performance indicators (KPIs). This led to participants feeling alienated from their peers and largely responsible to control the impact of the cyber incident as is shown in the examples corresponding to this heading in Table 5.

Participants also described normal office environments with open plan spaces and normal noise levels. They all described physical environments where they could concentrate on tasks. In our results physical environment did not seem to be a contributor to UIsT but it did not also mitigate the threat from occurring. These findings contributed to evaluating relationships between peers and monitoring attention to detail in virtual tasks and physical environment within our framework. Having strong relationships at a peer level showed to have positively influenced mitigating against UIsT.

#### External factors

When speaking about their personal methods of working participants shared certain traits that might have contributed to UIsT. The interviews uncovered specific individual traits within our participants that might have been stimulated through various external factors. The traits reflected by our participants included being willing to take on and expect ad hoc work, being responsible for multiple projects, anticipating workload, taking personal responsibility for the delivery of tasks assigned to them and being detail oriented. Participants also appeared to possess good communication skills and the ability to ask for help which allowed participants to reduce the impact of the cyber breach. These traits also reflect a deeper connection to external factors such as job security and losing income for the organisation. Whist there appeared to be a tension between prescribed processes and factors that might influence deviation, all our participants also showed an active commitment to best practices and compassion for others who inputted into their tasks. In our study participants appeared to compromise on prescribed processes in favour of compassion for others. Beyond compassion for others, our discussions with all participants showed self-esteem as another factor that participants were actively considerate of. This included how they appeared to themselves and others around them.

The above findings pertaining to external factors appeared to influence the conditions that facilitated an unintentional cyber breach. These findings contributed to inputs for assessing prioritization of processes, commitment to best practices, personal responsibility taken by individuals when delivering tasks and levels of stigma associated to near-misses or accidents within our framework. We will now discuss these in greater detail in the following section.

## Discussion

**Fig. 3:**
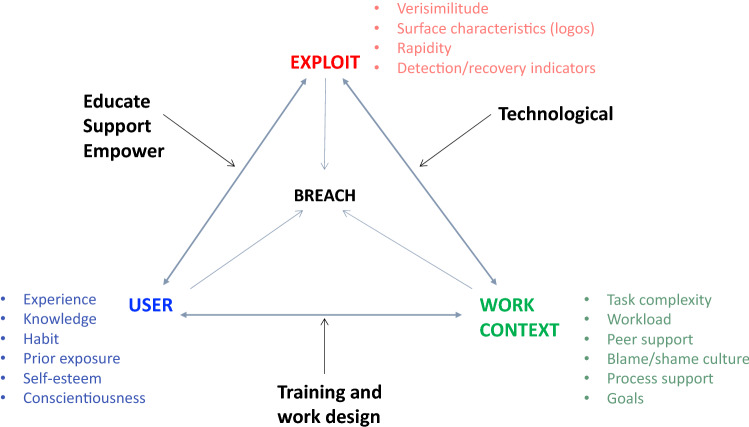
Summarising the results through an Epidemiological Triangle to represent the interplay between the three vectors of user, exploit and work context for UIsT

The findings from our study discussed in the results section above were characterised as either features or action suggestions. Examples of features included data codes such as suspect logos, deviation in language, speed, compassion for others while action suggestions encompassed actions, such as turning equipment off, performing tasks and subsequent task elements, rushing, being reprimanded amongst other codes. One way of summarising our results in an accessible form is to adopt the convention of the Epidemiological Triangle most commonly used in public health communication. Classically, the triangle (or triad) represents the interplay between the Host (the putative victim of an infection), the Agent (the disease itself) and Environment in which both exist. The Host (the User) and Agent (the Exploit) may have various forms of intrinsic resistance and virulence, respectively, which are strengthened or weakened relative to each other by the environment (the Work Context), shown in in Fig. 3. This represents three vectors that may be militated against to reduce the chance of an incident (e.g., by prophylactic measures that strengthen the host, anti-disease steps that weaken the agents or by modifying the environment). This approach has also been used in the context of safety science (e.g., Gordon 1949; Haddon 1968). Here, we position the probability of a breach in relation to the features of the Exploit itself, the qualities of the User and their prior experience and the Work Context in which the breach occurs.

The overview exhibited through the Epidemiological Triangle allowed us to gain a deeper understanding of who would need to take responsibility for different elements of intervention, providing a structure to delivering any interventions. Accordingly, data codes were re-classified to create a framework (shown in Table 7) which informs the following discussion. For the purposes of this work a framework is a set of recommendations applicable in specific scenarios to reduce negative impact. Our framework proposes a five-pillar action plan listed as Outputs that can be achieved through 35 distinct Inputs. Based on our findings Input elements can be captured to assess the potential level of risk in a setting and therefore provide appropriate timely interventions. We believe this framework can be implemented by organisations that are interested in starting an UIsT program or as an evaluation tool for organisations that currently have one. We advise that this framework is conducted bi-annually or when organisational changes occur. It is also worth noting that we recommend tailored trainings that goes beyond traditional face-to-face teaching and are audited for their effectiveness.

**Table 7:** Framework: each of the five outputs represent a pillar within the framework. Pillars encompass the elements that can be examined to indicate UIsT levels within an organisation and are informed by respective Inputs. These Inputs are variables that influence UIsT emerging from research study’s findings and involve various sociotechnical elements. This framework serves as a blueprint for identifying, intervening and mitigating UIsT and will inform future works that involve the representation and design of a tool that can be utilised by organisations

Outputs	Pillar no.
User vulnerabilities to UIsT and recommendations to strengthen defences	1
The effectiveness of processes and facilitating a continuous improvement culture	2
Workload and sufficient resource allocation	3
Knowledge sharing and empowerment culture	4
Fluctuating vulnerabilities	5

Inputs	Contributes to
Assess how comfortable individuals are with various technologies and platforms	Pillar 1
Assess how vulnerable users feel in their daily online interactions	Pillar 1
Assess physical working environments	Pillar 1
Assess individuals' ability to identify spear phishing scams to note vulnerabilities	Pillar 1
Assess individuals' existing experiences with malware or threats (including physical spaces)	Pillar 1
Assess individuals' knowledge base to evaluate understanding of current techniques used by hackers	Pillar 1
Assess individuals' susceptibility to rationalise abnormal behaviour or interactions	Pillar 1
Assess individuals' susceptibility to spear phishing	Pillar 1
Assess individuals' trust in technologies	Pillar 1
Assess the levels of how much individuals rely on their social networks (offline and online) to inform their decisions if faced with threats	Pillar 1
Assess individuals' awareness of mainstream marketing campaigns against popular attacks	Pillar 1
Assess levels of retention from basic ICT teachings to establish levels of awareness	Pillar 1
Assess and map different skill levels between individuals in a diverse workforce	Pillar 1
Assess individual's level of caution when interacting with suspicious or odd behaviour (online and physical parameters)	Pillar 1
Evaluate all tasks to identify missing feedback loops that indicate task completion	Pillar 1
Evaluate the effectiveness of prescribed processes amongst skilled/experienced staff	Pillar 2
Assess individuals' prioritization of processes	Pillar 2
Evaluate the effectiveness of prescribed processes amongst all designations	Pillar 2
Evaluate in-use software's limitations in prescribed processes	Pillar 2
Assess individuals' commitment to best practices set out by the company	Pillar 2
Evaluate processes for collaborative tasks that are automated	Pillar 2
Assess individuals' technical skill levels	Pillar 2
Assess individuals' levels of personal responsibility felt when delivering tasks assigned to them	Pillar 3
Assess resources available to individuals to deliver tasks	Pillar 3
Assess individuals' motivations when delivering tasks	Pillar 3
Assess individuals' ability and willingness to take on additional tasks	Pillar 3
Assess levels of stigma associated with experiences of near misses and accidents that result in cyber incidents and cyber breaches across all levels	Pillar 4
Assess levels of communication about cyber incidents	Pillar 4
Assess individuals' understanding of outcomes that result from accidents	Pillar 4
Evaluate effectiveness of current guidelines in the event of a cyber breach	Pillar 4
Evaluate individuals' understanding of protocols in the event of a cyber breach	Pillar 4
Evaluate individuals' ability to question, share and challenge abnormal interactions	Pillar 4
Evaluate relationships between individuals and managers across all levels	Pillar 4
Evaluate relationships between peers across all levels	Pillar 4
Assess individuals' level of attention to detail (online and physical parameter)	Pillar 5

### Technical defences

Passive cyber defences are undoubtedly a good measure to serve as the first line of defence to protect networks against attacks. This should include virtual and physical spaces encompassed in examples discussed in the background section earlier. While our framework lists a few technical defence elements, such as conducting penetration testing and mapping all staff’s ICT skillsets, it is recommended that all passive defences within technical defences are fully inclusive in the implementation of this framework. These include best practices for software architecture, monitoring user activities and devices, configuration, encryption, managing access points (including privileges), data management, updating software and regular audits. Passive defences are in-line with NCSC’s and CERT’s recommendations but in contrast to NCSC and CERT we do not recommend restriction of devices or features as it can encourage users to create unauthorised or unmonitored back channels for delivering tasks. Instead in the following section we propose evaluating and noting how tasks are conducted so suitable defences can be implemented. While it would be desirable to develop in-house technical skills through formal training as suggested by NCSC, as part of the first pillar we recommend mapping existing skills to identify talent that can be utilised and developed across all designations. We also do not recommend the use of active cyber defences such as those used in SOFIT. Active defences can create complexities for implementation as they are dependent on permissibility in local laws (such as packet sniffing) as they infringe on individual privacy and can foster distrust between the organisation and individuals. Active defences also do not appear to be effective for safeguarding against UIsT as active monitoring against unintentional actions is fundamentally inapplicable.

### Sociotechnical defences

Points contributing to each outcome in our framework are not separated as belonging to individual, technological or organisational contexts but rather findings are integrated to provide effective solutions that can identify, intervene and mitigate UIsT. The framework lists inputs which provide objectives for evaluations and outputs which are the five pillars to help gauge levels of vulnerability to UIsT and provide recommendations to strengthen defences. The framework introduces ‘Stop, Think, Ask, Action, Consequence’ or STAAC that is used to foster Type 2 thinking that counteracts UIsT. STAAC can be used prior, during or after a threat has been realised.

#### User vulnerabilities to UIsT and recommendations to strengthen defences

The first pillar of our framework provides an assessment report to benchmark existing vulnerabilities to UIsT within an organisation. This is done by evaluating 15 distinct points listed as inputs in Table 7.

In contrast to CERT, SOFIT and other popular models that rely on psychological and behavioural profiling our framework does not assign methods or people for evaluating elements within inputs. As this framework specifically targets UIsT we also do not require any individuals (such as HR personnel or peers) deducing individual personality traits or reporting suspicious behaviours that are geared towards identifying intentional IsT. Instead to benchmark vulnerabilities we evaluate individual’s comfortability with various technologies, risk awareness levels (in-line with NCSC), individual lived experience and acquired levels of knowledge to identify malicious content and individual susceptibility to rationalise anomalies in interactions. We also assess physical environment for environmental stressors that are indicated in SOFIT and individual’s trust in technologies to safeguard against malicious content. As part of the actions to evaluate UIsT, it encompasses educating and raising awareness amongst individuals through traditional and hands-on training as suggested by NCSC and CERT.

#### The effectiveness of processes and facilitating a continuous improvement culture

Our results support a link between task processes and the risk of breaches and validates Liginlal et al.’s (2009) recommendation for developing effective processes to tackle UIsT. In addition to effective processes our findings highlighted the importance of individuals understanding why steps within a process are important. For cases where explicit processes existed, our participants who had good expertise at performing their tasks, skipped steps as the importance of following each step was not communicated and had resulted in near-misses than accidents in the past but never an actual breach.

In this section of the framework, we recommend the evaluation of processes with the help of staff who possess good expertise for performing their assigned tasks. In contrast to Liginlal et al. who focus on addressing the lack of expertise through training, we emphasize the importance of working with expert individuals to identify heuristics and shortcuts that can facilitate UIsT. It also allows creation of processes that reflect ‘work as done’ as opposed to ‘work as imagined’ (Hollnagel 2017, Suchman 1985). At this stage we also assess the processes’ effectiveness, individual prioritisation techniques that might compromise processes, conducting task analysis to device effective and improved processes as the delivery of the task changes, evaluating tasks that include automated elements and mapping software limitations that foster undesirable practices being implemented. While it is important to carefully consider implementing new systems that can facilitate new errors (Liginlal et al. 2009), it is also critically important to evaluate existing systems’ effectiveness and suitability for prescribed processes. As part of this stage, we also evaluate individuals’ commitment to best practices, trade-offs made and mapping individual technical skill levels when delivering tasks as all these factors were shown to influence UIsT.

#### Workload and sufficient resource allocation

Assessment of workload and allocating sufficient resources is the third pillar of this framework. SOFIT (Greitzer et al. 2018) includes workload as an indicator and while time pressures are the cornerstones for CERT and Nurse et al.’s frameworks for indicating UIsT our results highlighted additional interlinked factors. These factors included individuals feeling personally responsible for delivering tasks and allocation of sufficient resources (which included time and people) which is discussed in greater detail in the previous section. Individuals’ motivations for delivering tasks are also important to consider as motivations such as rushing or meeting unrealistic deadlines (in-line with SOFIT) can support human fallibility. In addition, our framework incorporates organisational expectation for individuals to undertake new tasks that were also seen to be linked to UIsT.

In contrast with SOFIT, our results did not suggest strong links between UIsT and poor team management as participants reportedly enjoyed good interpersonal relationships with their direct line managers and peers. We did not find evidence for the mismatch between expectations and abilities listed in Liginlal et al.’s framework as participants had mid to advanced level of expertise to perform assigned tasks. We also did not find any evidence to support ambiguous goal setting or poor communication of goals (no data gathered for poor morale) all of which are factors included in SOFIT. Therefore, these elements have not been included in the framework.

#### Knowledge sharing and empowerment culture

NCSC, CERT, Liginlal et al. and SOFIT all include blame culture as being an indicator of UIsT and recommend instilling a culture of empowerment to counteract IsT. Our results also found that alongside an empowerment culture, which influenced UIsT, how knowledge was availed and shared between individuals had an impact on UIsT risks.

This section of the framework evaluates the culture of an organisation through assessing stigma and levels of organisational communication associated to cyber incidents and breaches. It also evaluates individual’s understanding of outcomes that are associated to cyber breaches, effectiveness of guidelines in the event of an attack and individual understanding of subsequent protocols, ability to challenge abnormal interactions and inter-organisational relationship dynamics. We recommend creating security protocols based on STAAC. As UIsT is rapid in its nature and participants noticed anomalies that they ignored we introduce STAAC to assist individuals in slowing down and thinking through their actions at various points in the cyber breach (prior or during) which can help identify, intervene and mitigate UIsT. As suggested by NCSC, our framework also promotes the importance of practicing cyber breach protocols through drills (incorporating STAAC) and establishing an incident reporting culture through creating platforms (off-line and online) for knowledge sharing.

#### Fluctuating vulnerabilities

As the final pillar and further to the relevant frameworks discussed above, we propose a regular assessment of fluctuating vulnerabilities discussed above that can influence UIsT. As UIsT is changing in its nature due to these fluctuating vulnerabilities, we suggest using several indicators, such as evaluating attention to detail (online and physical parameter), to formulate recommendations.

## Conclusions, limitations and future work

As attacks get increasingly sophisticated, well-intentioned employees have become prime targets for hackers to enable successful cyberattacks such as ransomware. UIsT continues to constitute a major threat to organisational assets that can result in significant disruption to operations as well as substantial financial and reputational damages. In order to tackle this threat effectively organisations need to have a clear understanding of UIsT and factors that influence it in complex environments. In this paper we discussed UIsT as a separate and distinct phenomenon to intentional IsT to build a better understanding of this threat. We also proposed an amalgamated framework developed through grounded theory applied to interview datasets and further refined through the Epidemiological Triangle.

As a result of our findings the framework incorporated new findings and enhanced existing elements from relevant frameworks designed to tackle IsT (intentional and UIsT). Existing elements that are utilised in our framework include the use of passive defences (NCSC and CERT), mapping in-house technical skills across all designations to build talent (NCSC), risk awareness (NCSC), evaluating physical environmental stressors (SOFIT), educating and raising awareness through training (NCSC and CERT), evaluating processes (Liginlal et al.), monitoring time pressures (CERT and Nurse et al.) and instilling an organisational culture of empowerment (NCSC, CERT, Liginlal et al., SOFIT). New features introduced in this framework include the following:

- Listing of rationale behind steps within processes, designing and evaluating processes with staff who possess expertise at performing assigned tasks and periodically evaluating processes’ effectiveness through ‘work as conducted’ are essential
- New interlinked factors to time pressures are incorporated
- Knowledge attainment and sharing in addition to an empowerment culture is recommended
- UIsT involves fluctuating vulnerabilities that must be known, monitored and addressed

We envisage that the framework presented in this paper at Stage 1 could be adopted by small and large organisations alike to build and strengthen effective sociotechnical defences against cyber breaches stemming from UIsT. STAAC is introduced as a guiding principle to help individuals identify, intervene and mitigate against UIsT through Type 2 thinking which is embedded through regularly audited trainings, workshops and drills. Our Stage 1 framework sets out five pillars listed as outcomes which might be achieved through the evaluation of various Inputs that appear to influence UIsT.

Limitations of this work emerge from snowball sampling that might limit the diversity of participants and in turn the generalisability of the findings. Some participants were already known to the interviewer; this facilitated candid and honest discussions, but this rapport might have also influenced participants’ responses to some degree. As participants were asked to recall an incident in the past, whilst this incident was significant in their lived experiences, the application of recall and memory bias might unintentionally include or exclude information that might be significant for the findings.

Future directions stemming from the research presented in this paper are currently being explored. This includes the adoption of the framework at an organisation(s) for validation. Adoption of the framework in industry settings can provide a measure of effectiveness of the suggestions through incident rates prior and post adoption. It can also provide an opportunity to collect feedback from end users about the design and display of outputs (UX). Future work might also involve the creation of recommendations (what steps to take) that might strengthen inputs if they are reportedly weak and in turn strengthen pillars and ultimately defences against UIsT whilst maintaining human agency and empowerment within the system.

## References

[CR1] Agrafiotis I, Nurse JCR, Buckley O, Legg P, Creese S, Goldsmith M (2015). Identifying attack patterns for insider threat detection. Comput Fraud Secur.

[CR2] Ani U, Daniel N, Oladipo F, Adewumi S (2018). Securing industrial control system environments: the missing piece. J Cyber Secur Technol.

[CR3] Bearman C, Bremner P (2013). A day in the life of a volunteer incident commander: errors, pressures and mitigating strategies. Appl Ergon.

[CR4] Bell A, Rogers M, Pearce J (2019). The insider threat: behavioral indicators and factors influencing likelihood of intervention. Int J Crit Infrastruct Prot.

[CR5] Bhaskar R (1989). Reclaiming reality: a critical introduction to contemporary philosophy.

[CR6] Bishop M, Engle S, Peisert S, Whalen S, Gates C (2008) We have met the enemy and he is us. In: Proceedings of the 2008 new security paradigms workshop. 10.1145/1595676.1595678

[CR7] Canham M, Posey C, Bockelman P (2020). Confronting information security’s elephant, the unintentional insider threat. Int Conf Hum Comput Interact HCI.

[CR8] Cappelli D, Desai A, Moore A, Shimeall T, Weaver E, Willke B (2007). Management and education of the risk of insider threat (MERIT): mitigating the risk of sabotage to employers information, systems, or networks. Carnegie Mellon Univ.

[CR9] Cappelli D, Desai A, Moore A, Shimeall T, Weaver E, Willke B (2008) Management and education of the risk of insider threat (MERIT): system dynamics modeling of computer system. Carnegie Mellon University, Pittsburgh. https://resources.sei.cmu.edu/library/asset-view.cfm?assetid=52324. Accessed 17 Sep 2020

[CR10] CERT Insider Threat Team (2013). Unintentional insider threats: a foundational study. Softw Eng Inst.

[CR11] Chattopadhyay P, Wang L, Tan Y-P (2018). Scenario-based insider threat detection from cyber activities. IEEE Trans Comput Soc Syst.

[CR12] Dice Staff (2020) Cybersecurity in 2021: 5 Trends Security Pros Need to Know. Dice Insights. https://insights.dice.com/2020/12/14/cybersecurity-in-2021-5-trends-security-pros-need-to-know/. Accessed 17 Dec 2020

[CR13] Evans JSBT (2012). Spot the difference: distinguishing between two kinds of processing. Mind Soc.

[CR14] Glaser BG, Strauss AL (1967). The discovery of grounded theory: strategies for qualitative research.

[CR15] Goethals PL, Hunt ME (2019). A review of scientific research in defensive cyberspace operation tools and technologies. J Cyber Secur Technol.

[CR16] Gordon J (1949). The epidemiology of accidents. Am J Public Health Nations Health.

[CR17] Greitzer FL, Hohimer RE (2011). Modeling human behavior to anticipate insider attacks. J Strateg Secur.

[CR18] Greitzer F, Purl J, Leong YM, Becker DES (2018) SOFIT: sociotechnical and organizational factors for insider threat. In: 2018 IEEE security and privacy workshops. 10.1109/SPW.2018.00035

[CR19] Haddon W (1968). The changing approach to the epidemiology, prevention, and amelioration of trauma: the transition to approaches etiologically rather than descriptively based. Am J Public Health Nations Health.

[CR21] Hadlington L (2018) The “human factor” in cybersecurity: exploring the accidental insider. In: McAlaney J, Frumkin LA, Benson V (eds) Psychological and behavioral examinations in cyber security. IGI Global, pp 46–63. 10.4018/978-1-5225-4053-3.ch003

[CR20] Hoda R, Noble J, Marshall S (2010). Using grounded theory to study the human aspects of software engineering. HAoSE.

[CR22] Hoffman RR, Crandall B, Shadbolt N (1998). Use of the critical decision method to elicit expert knowledge: a case study in the methodology of cognitive task analysis. Hum Factors.

[CR23] Hollnagel E, Wears RL, Braithwaite J (2015) From Safety-I to Safety-II: a white paper. University of Southern Denmark, University of Florida and Macquarie University. 10.13140/RG.2.1.4051.5282

[CR24] Hollnagel E, Wears RL, Hollnagel E (2017). Why is work-as-imagined different from work-as- done?. Resilient health care.

[CR25] Hunker J, Probst C (2011) Insiders and insider threats—an overview of definitions and mitigation techniques. J Wirel Mob Netw Ubiquitous Comput Dependable Appl. 10.22667/JOWUA.2011.03.31.004

[CR26] Kammüller F, Probst CW (2013) Invalidating policies using structural information. In: 2013 IEEE security and privacy workshops. 10.1109/SPW.2013.36

[CR27] Keeney M, Kowalski E, Cappelli D, Moore A, Shimeall T, Rogers S (2005) Insider threat study: computer system sabotage in critical infrastructure sectors. National Threat Assessment CTR, Washington. https://apps.dtic.mil/dtic/tr/fulltext/u2/a636653.pdf. Accessed 23 Sep 2020

[CR28] Klein GA, Calderwood R, MacGregor D (1989) Critical decision method for eliciting knowledge. In: IEEE transactions on systems, man, and cybernetics. 10.1109/21.31053

[CR29] Legg PA, Buckley O, Goldsmith M, Creese S (2017). Automated insider threat detection system using user and role-based profile assessment. IEEE Syst J.

[CR30] Liginlal D, Sim I, Khansa L (2009). How significant is human error as a cause of privacy breaches? An empirical study and a framework for error management. Comput Secur.

[CR31] Magklaras G, Furnell S (2002). Insider threat prediction tool: evaluating the probability of IT misuse. Comput Secur.

[CR32] Morel B (2011) Artificial intelligence and the future of cybersecurity. In: Proceedings of the 4th ACM workshop on security and artificial intelligence (AISec '11), Association for Computing Machinery, New York. 10.1145/2046684.2046699

[CR33] Mundie DA, Perl S, Huth CL (2013) Toward an ontology for insider threat research: varieties of insider threat definitions. In: 2013 third workshop on socio-technical aspects in security and trust. 10.1109/STAST.2013.14

[CR34] Muller MJ, Kogan S (2010). Grounded theory method in HCI and CSCW.

[CR35] NCSC (2012) 10 steps to cyber security: guidance on how organisations can protect themselves in cyberspace, including the 10 steps to cyber security. https://www.ncsc.gov.uk/collection/10-steps-to-cyber-security. Accessed 27 Aug 2020

[CR36] Neal A, Griffin MA (2004) Safety climate and safety at work. In: Barling J and Frone MR (eds) The psychology of workplace safety. American Psychological Association, pp 15–34. 10.1037/10662-002

[CR37] Norman DA, Norman DA, Draper SW (1986). Cognitive engineering, chapter 3. User centered system design; new perspectives on human-computer interaction.

[CR38] Nurse JRC, Buckley O, Legg PA, Goldsmith M, Creese S, Wright GRT, Whitty M (2014) Understanding insider threat: a framework for characterising attacks. In: 2014 IEEE security and privacy workshops. 10.1109/SPW.2014.38

[CR39] Ogiela MR, Ogiela U (2012). Linguistic protocols for secure information management and sharing. Comput Math Appl.

[CR40] Pauley K, Flin R, Yule S, Youngson G (2011). Surgeons' intraoperative decision making and risk management. Amjsurg.

[CR41] Plant KL, Stanton NA (2013). What is on your mind? Using the perceptual cycle model and critical decision method to understand the decision-making process in the cockpit. Ergonomics.

[CR42] Predd J, Pfleeger SL, Hunker J, Bulford C (2008). Insiders behaving badly. IEEE Secur Priv.

[CR43] Reason J (1998). Achieving a safe culture: theory and practice. Work Stress.

[CR44] Reason J, Manstead A, Stradling S, Baxter J, Campbell K (1990). Errors and violations on the roads: a real distinction?. Ergonomics.

[CR45] Schuh G, Potente T, Wesch-Potente C, Weber AR, Prote JP (2014). Collaboration mechanisms to increase productivity in the context of Industrie 4.0. Proc CIRP.

[CR46] Siegel H (2004) Relativism. In: Niiniluoto I, Sintonen M and J Wolenski (eds) Handbook of Epistemology. Springer, Dordrecht, pp 747–780

[CR47] Suchman L (1985). Plans and situated actions: the problem of human–machine communication.

[CR48] Vanderhaegen F, Wolff M, Mollard R (2020). Non-conscious errors in the control of dynamic events synchronized with heartbeats: a new challenge for human reliability study. Saf Sci.

[CR49] Verizon (2020) Data Breach Investigations Report. https://enterprise.verizon.com/resources/reports/2020-data-breach-investigations-report.pdf. Accessed 12 Jan 2021

[CR50] Wong BLW, Diaper D, Stanton N (2004). Critical decision method data analysis. The handbook of task analysis for human–computer interaction.

[CR51] Woods DD, Hollnagel E (2006). Joint cognitive systems: patterns in cognitive systems engineering.

[CR52] Zargar A, Nowroozi A, Jalili R (2016) XABA: a zero-knowledge anomaly-based behavioral analysis method to detect insider threats. In: 2016 13th International Iranian society of cryptology conference on information security and cryptology (ISCISC). 10.1109/ISCISC.2016.7736447

